# A Critical Appraisal of New Developments in Intraocular Lens Modifications and Drug Delivery Systems for the Prevention of Cataract Surgery Complications

**DOI:** 10.3390/ph13120448

**Published:** 2020-12-08

**Authors:** Ioanna Mylona, Ioannis Tsinopoulos

**Affiliations:** 2nd Department of Ophthalmology, Aristotle University of Thessaloniki, 564 29 Thessaloniki, Greece; itsinop@med.auth.gr

**Keywords:** intraocular lens, cataract, drug delivery

## Abstract

Cataract surgery is the commonest ophthalmic surgery worldwide. The replacement of the diseased lens with a synthetic one (intraocular lens—IOL) remains the treatment of choice, despite its potential complications that include infection, inflammation and posterior capsule opacification. The potential for drug delivery via the IOL has been researched extensively over a period of twenty-five years, yet there is very limited progress in transferring the findings from research to everyday practice. The objective of this review is to assess the progress made in the field of IOL lens modifications and drug delivery systems over the past five years. Thirty-six studies that were conducted during the past five years were identified and deemed suitable for inclusion. They were grouped in three broad categories, studies that described new methods for loading a drug onto the IOL, assessment of the effects of drugs that were loaded to the IOL and studies that assessed the effects of non-pharmaceutical modifications of IOLs. While considerable progress is continually being made with regard to methods and materials, there is still little capitalization upon these research studies, with no commercially available IOL-based drug delivery system being available. Close cooperation between researchers in basic sciences (chemistry, physics, materials science and pharmacy), clinical researchers, IOL manufacturers and the pharmaceutical industry is an important prerequisite for further development.

## 1. Introduction

The eye lens is a transparent biconvex structure comprised of fiber cells and crystallins, proteins that are critical to maintaining its high refractive index. The lens refracts light to be focused on the retina and produce sharp vision. It consists of a capsular bag and the lens substance, which is separated from the capsular bag by a monolayer of lens epithelial cells (LECs) on its anterior surface [1]. Cataract, an opacification of the native lens due to ageing, cardiovascular and metabolic disease or various obnoxious environmental factors, remains the most significant cause of reversible blindness in the world [2] with congenital and infantile cataract responsible for a fifth of the world’s blind children [3] and secondary cataract due to trauma or previous ophthalmic surgery adding a significant additional burden [4]. Since the 1990s, cataract surgery has progressed to the introduction of the modern technique of phacoemulsification along with new local anesthesia mediums. Phacoemulsification involves a small corneal incision and the replacement of the native damaged lens with a polymer-based substitute, an artificial intraocular lens (IOL) to restore unhindered vision, with cataract surgery considered among the most successful treatments in all of medicine [5]. A related advancement is in the field The first IOL was created in 1949 by Ridley from poly(methyl methacrylate) (PMMA) [6]. Subsequently, the use of hydrogels to make flexible and foldable IOLs has alleviated the disadvantages of large incision requirement, iris atrophy, and glaucoma, and thus greatly reduced the wound healing time. The next generation of IOLs were siloxane-based hydrogels that could be folded to assist with its insertion. The latest developments are hydrophilic hydrogels and hydrophobic acrylate polymer lenses which, coupled with new local anesthesia mediums, reduce intraoperative and perioperative complications, reduce the subjective experience of pain and associated costs [7,8].

Unfortunately, although lens replacement surgery has high success rates, there are also adverse postoperative outcomes that include posterior capsule opacification (PCO), intraocular inflammation and infection (endophthalmitis) while more rare postoperative complications are retinal detachment, acute serous macular detachment and corneal decompensation or edema after uncomplicated phacoemulsification, leading to efforts aiming to assess the preoperative risk [9]. Additionally, several complications can occur more frequently during a secondary IOL implantation, when the risk of inflammation is even higher. This risk can also vary according to the invasiveness of the technique used (sutureless or not, anterior-chamber fixated or posterior-chamber fixated, etc.) [10]. This wide range of complications are potential therapeutic targets for an appropriately modified IOL, justifying the considerable research interest in the field.

PCO in particular is a proliferation of remaining epithelial cells from the original lens on the posterior end of the IOL that ultimately leads to light scatter and to a reduction in visual acuity [11]. PCO is more frequent in younger age at the time of surgery, diabetes, lens nucleus hardness of III–V grade, extracapsular cataract extraction, post-vitrectomy, and hydrophilic IOLs with a recent study of 652 eyes from 550 patients with a three-year follow-up determined that there were 108 eyes (16.5%) with PCO [12]. These data are comparable to results from a meta-analysis of more than twenty years ago that showed incidence of PCO as 11.8% (9.3–14.3%) at 1 year, 20.7% (16.6–24.9%) at 3 years, and 28.4% (18.4–38.4%) at 5 years after surgery [13]. This persistence of the high incidence despite the progress in materials and surgical technique over those twenty years demonstrates the importance of finding new ways to combat potential PCO and while a non-invasive YAG laser procedure can safely resolve the issue through PCO capsulotomy, this however is an expensive treatment with its own set of potential complications; a recent long-term observation study found the incidence of retinal detachment was 0.87% at five months post Nd:YAG, while the rate of retinal tears after Nd:YAG capsulotomy at five months was 0.29% [14].

### IOL Modification to Prevent Adverse Outcomes in Cataract Surgery

Researchers have developed IOL material modifications in order to prevent adverse outcomes of cataract surgery during the past forty years [15]. In particular, attempts to integrate active molecules to an IOL for the same goal, have been ongoing for close to twenty-five years, following the pioneering work by Nishi et al. [16] and Tetz et al. [17]. The only commercially available method to address potential cataract surgery complications is the administration of eye drops post-operatively. This presents a significant problem in that the majority of postoperative cataract patients are inexperienced with eye drop use and show a poor instillation technique, as reported in a study by An et al. [18]. Hence, a new medium of postoperative drug delivery is necessary in order to optimize the results. The development of a modified IOL to act as a drug delivery device has been pursued for many years and is a promising concept in modern cataract surgery.

**Figure 1:**
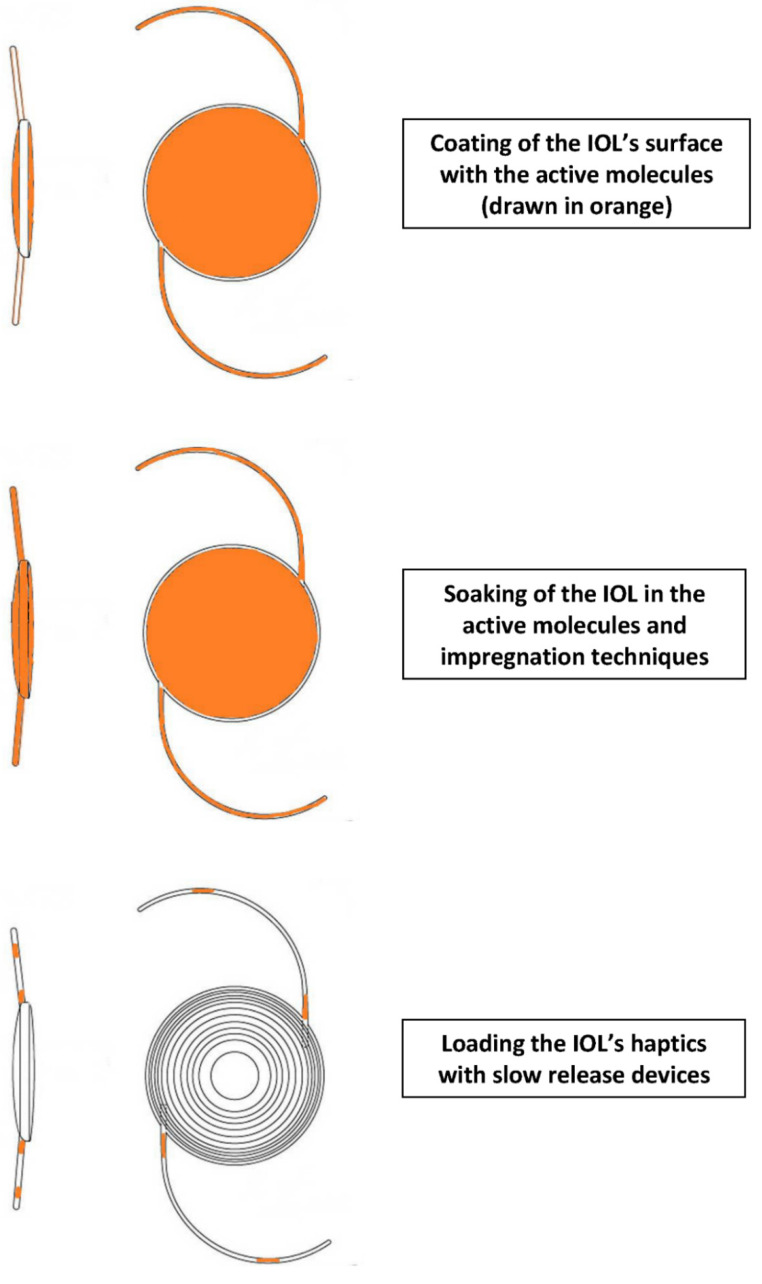
Graphical representation of modes for enabling drug delivery via an intraocular lens (IOL).

There are three techniques of pharmacological IOL modification. The most frequent is surface modification of the IOL. This can be achieved by impregnation with supercritical fluids or chemically coating with polymer/drug layers, (“coating”), or by coating the lens surface with PEG or ‘‘bioactive’’ polymers exhibiting sulfonate and carboxylate groups. Theses successfully prevent cell adhesion and proliferation [19,20]. Another IOL modification is by way of immersion in high drug concentration solutions (“soaking”). Finally, the most recent development involves loading the IOL haptics with a slow release system [21]. Figure 1 presents a graphic representation of those three techniques.

Haptics are additional IOL components that keep them in place in the capsular bag. These haptics can be utilized for drug delivery, with the added benefit of not directly supporting vision with any optical qualities of their own, and are not subject to inherent limitations of the rest of the IOL. This is an important detail, since an alternative of a drug-eluting contact lens is typically limited in its drug-loading potential by the need for complete transparency. For example, the haptics can be coated with non-transparent polymers, drug suspensions, or molecules that would otherwise reduce the optical properties of the lens material [22]. Thus, a drug-eluting IOL can reduce the risk of endophthalmitis and postoperative infection related to cataract surgery, but also deliver drugs to other regions of the eye [22]. Such drug-loaded IOLs may have potential benefits in the prevention of endophthalmitis, a frequent complication of cataract surgery; they may prevent postoperative inflammation and reduce the risk for PCO. There are certain prerequisites however that still need to be adhered to; the agent must be toxic to the LECs while not having any adverse effects on the corneal endothelium. An ideal refinement of this method of delivery would be capable of releasing the molecule either directly from the lens itself or from the haptic, at a steady low pace following an initial burst to achieve therapeutic levels. Thus, we may ensure sufficiently high concentrations to affect the LECs but nontoxic to the corneal endothelial cell layer.

Two major issues in the early efforts for a viable IOL drug release system were the timeframe for the drug release and the potential toxicity of the drug, either due to its nature or due to its initial concentration after the release. Postoperative endophthalmitis is an eye infection of exogenous origin and its onset may be acute or delayed (chronic). The acute form develops within six weeks after surgery, with signs and symptoms in patients occurring within the first two weeks, typically within 4–7 days, while the chronic form develops six weeks or more after surgery [23]. A standard prevention strategy is to decrease or eliminate eyelid and conjunctival microflora both preoperatively and intraoperatively. This goal is typically served by using preoperative topical antibiotics and topical antiseptic agents, while subconjunctival antibiotics may be administered at the time of surgery. Studies of drug-eluting IOLs loaded with antibiotics typically aim to cover the two-week window of an acute-onset, post-operative infection. Chronic endophthalmitis is typically attributed to *Propionibacterium acnes* that has many drug-resistant strains [23] and fungi; the best prevention strategy is topical antiseptic agents prior to surgery since the response to antibiotics is poor.

The question of minimum time requirement for PCO prevention has not been conclusively resolved due to our gaps in knowledge regarding the pathophysiology of PCO itself; the proliferation of LECs depend on the interaction between a variety of cytokines including fibroblast growth factor (FGF) and transforming growth factor β (TGFβ). FGF increases after surgery and stimulates proliferation of LECs while the initially inactive TGFβ is gradually activated and induces epithelial mesenchymal transition of LECs, leading to production of extracellular matrix. TGFβ levels are likely to remain elevated in the ocular environment for days or weeks following surgery, thus extending the timeframe for PCO prevention; the reader is referred to in a recent review by Wormstone et al. for an exhaustive presentation of the issue [11]. In most instances the therapeutic window for drug release was ten days, sufficient enough to treat a bacterial infection following cataract surgery but insufficient for addressing PCO [24].

A separate issue that needs to be addressed is that the IOL also needs to be foldable, since a rigid one requires larger surgical incisions for its implantation, which could itself augment PCO; this was an issue with a method put forward by Siqueira et al. [25]

Therefore, the hurdles of the clinical application of a drug-loaded IOL require the assessment of a broad spectrum of pharmacological agents not just in preclinical studies but also in clinical studies that would determine the most suitable combination of the agent with the specific type of the IOL. While a small number of biotechnological manufacturers are pursuing the manufacture of an IOL drug delivery system, no commercially available product has materialized yet.

## 2. Results

**Figure 2:**
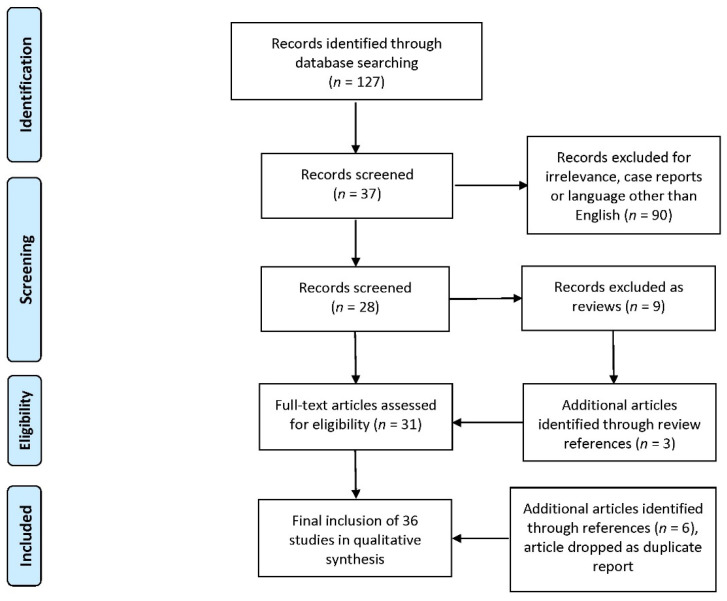
Flowchart of study selection according to PRISMA guidelines.

The search string described in the Materials and Methods section returned 127 manuscripts; 90 of those were either related to different disciplines or targeted other conditions or didn’t fit the inclusion criteria and were dropped. Nine of the remaining were review papers and not included in the study, although their references were screened for additional sources revealing three additional manuscripts. This brought the total number of selected papers to thirty-one, that were downloaded and assessed for their eligibility. Following further reference screening on those papers, six more were assessed while a paper that duplicated findings from the same study was rejected. The final selection of thirty-six papers that were included in the study was made independently by both authors and the decisions coincided. Figure 2 presents the selection process flowchart, according to PRISMA guidelines [26].

The selected studies can be broadly classified in three categories: (A) fourteen studies that present new improvements in modalities designed to assist the introduction of active molecules into the eye via an IOL, (B) seven studies that examine the therapeutic impact of specific drugs in the prevention of adverse outcomes following IOL implantation and (C) fifteen studies that present non-pharmacological modifications to IOLs in order to prevent inflammation, infection and PCO.

## 3. Discussion

### 3.1. Studies That Present New Improvements in Modalities Designed to Assist the Introduction of Active Molecules into the Eye via an IOL

Significant progress is being made in new methods for loading an IOL with active molecules. Fourteen studies have been published over the past five years that detail those methods or assess their efficiency. A detailed list of those articles is presented in Table 1.

For an effective surface modification, auxiliary substances, so-called linker molecules such as PLGA (polylactide-*co*-glycolide) or pHEMA (poly-2-hydroxyethyl methacrylate) may be employed to increase adsorption of hydrophilic pharmacological agents to the lens material but more importantly, as a hydrogel, pHEMA is also a suitable drug carrier (with new biodegradable derivatives now available [41]) and is currently being investigated for the loading and release of antibiotics for endophthalmitis prophylaxis [42]. In previous efforts, Bozukova et al. did not find any toxicity or other adverse effects when they chemically coated IOL surfaces with varying molecular weight PEG-chains to prevent both PCO and bacterial adsorption [20] while Anderson et al. [43] modified pHEMA in order to deliver norfloxacin to combat endophthalmitis. Those attempts were successful at maintaining adequate rate of release for five days, a timeframe that is sufficient for effective prevention of infection. Four recent studies are added to support those findings; Kassumeh et al. [33] spray-coated hydrophilic and hydrophobic acrylic IOLs with MTX-loaded polylactic-*co*-glycolic (PLGA) comparing them to unsprayed, solvent only, and solvent-PLGA-sprayed IOLs, in an in vitro study. Results showed a strong inhibition of PCO in vitro with the lack of toxic effects on a corneal cell line. Artigas et al. studied the intra-ocular lens optical changes resulting from the loading of dexamethasone using prototype hydrogel (pHEMA)-silicone IOLs [27]. The researchers examined the impact on MTF, spectral transmission and diopter power using a three-group experimental design that included drug-loaded IOLs, IOLs that had released the drug, and IOLs that had not been drug-loaded. Results indicating a transitory impairment of the MTF and spectral transmission in loaded IOLs that is almost recovered after the drug has been released. Dioptric power remained unimpaired; thus, the patient may be alerted to expect a gradual improvement of his eyesight over the course of the following days. Vieira et al. [40] examined in a similar study the effects from loading MFX on pHEMA-silicone IOLs by assessing morphology of sample cross-sections and the topography, coating thickness, refractive index and transmittance of visible light through the hydrated samples. They evaluated the in vitro antibacterial activity against *Staphylococcus aureus* and *Staphylococcus epidermidis* and cytotoxicity. The authors presented the characterization of the surface modification and found in studies of cytotoxicity that during five days there were no signs of an overt decrease in cell viability after contact with modified IOLs or with modified IOLs releasing MFX. The decrease in cell viability was well below the 30% threshold of a cytotoxic effect. This was the sole study that attempted to assess the effect on the MFX release profile of long soaking/storage times in an MFX solution, a storage study was undertaken employing the most promising system for periods of time ranging from 24 h up to 3 months, at room temperature. The authors found that while there was no case of drug loss, an increase in storage time in the MFX solution lead to an increase in the amount of MFX released and an increase in the release duration. The authors speculated that a very long loading time would allow drug molecules to reach much deeper into the IOL and longer to be released from the IOL surface.

Tan et al. tested moxifloxacin (MFX) and levofloxacin loadings to a degradable poly(l-lactide-*co*-ε-caprolactone) polymer multilayer film that acted as a depot and spray-coated to a commercially available IOL and haptic [38]. They selected tetrahydrofuran (THF) as the optimal solvent and the optimum IOL-attachment composition was between 3% to 5% of levofloxacin in a biodegradable PLC matrix, providing a release of levofloxacin at the required therapeutic doses over 14 days, to reduce the risk of infection.

The technique of “supercritical fluid” impregnation could prove to be particularly interesting [44]. A supercritical substance can flow through solids like a gas and dissolve materials like a liquid. This could result in a promising platform for the manufacture of drug carrier IOLs. Previously, Gonzalez-Chomon used supercritical fluid loading to improve the release characteristics of norfloxacin from 2-hydroxyethylmethacrylate-*co*-butoxy-ethylmethacrylate materials, showing release over 48 h although with high levels of burst in the initial period [45]. New developments in this field were demonstrated in two papers by Bouledjouidja et al. [28,29] and in a paper by Ongkasin et al. [35]. An important hurdle in this method is the appearance of undesirable foaming issues so as to obtain transparent IOLs. Bouledjouidja et al. [28] attempted to overcome this by coupling slow pressurization and depressurization phases during supercritical treatments of foldable IOLs made from pHEMA that were impregnated with the antibiotic ciprofloxacin and the anti-inflammatory drug dexamethasone 21-phosphate disodium (DXP). The authors tested ethanol as a co-solvent and determined it provided benefit only in the case of DXP impregnation. Release studies showed deep and reproducible impregnation for different diopters. The optical characterization of the lenses, including diopter power and modulating transfer function (MTF) values, were detailed in a separate paper confirming the preservation of optical properties after supercritical treatment/impregnation [29]. The combined conclusion from this research team was positive. Ongkasin et al. [35] expanded this knowledge base by impregnating IOLs with methotrexate in order to mitigate PCO in a case-control study. The results were favorable as there was a sustained release of methotrexate for more than 80 days and a reduction of fibrosis by the inhibition of epithelial-mesenchymal transformation. The use of ethanol as a co-solvent did not increase the impregnation efficiency and was even unfavorable at certain conditions, duplicating the negative result with ciprofloxacin in the Bouledjouidja et al. study [28].

A different method is the application of thin multilayers directly on IOLs as in the Han et al. study where the authors modified the IOLs with an antiproliferative drug-loaded hydrophilic chitosan coating [31]. The in vitro results showed that the proliferation of the residual lens epithelial cells on the IOL were inhibited while the in vivo animal experiments additionally showed excellent intraocular biocompatibility with adjacent tissues. The authors apparently report the same results in two published papers, one of them being a rapid communication [46]; thus the rapid communication was excluded from this review at the stage of examining for eligibility. The possibility of combining an active medication with chitosan was further studied by Sahariah et al. [37]; the authors employed three photoactive chitosan derivatives in a preliminary study of photografting and photocrosslinking of a polymethacrylate copolymer used industrially in the production of IOLs. The photografted polymethacrylate was loaded with MFX, and drug release profiles of the unmodified and photografted copolymer were evaluated and compared. The authors reported an optimized procedure for synthesizing chitosan derivatives of high molecular weight and determined the best performing derivative in terms of highest amount of released drug. In related developments, a research team led by Lambrogiannis [34] and Karamitsos [32] presented the results of an effort to design and fabricate of drug-eluting polymeric thin films. The researchers examined the characteristics of one- and two-layered thin films based on organic polymers [poly(d,l-lactide-*co*-glycolide) (0.65:0.35 w), poly(d,l-lactide-*co*-glycolide) (0.75:0.25 w), and polycaprolactone] and dexamethasone. Those thin films were prepared by the spin-coating method on a silicon substrate and dexamethasone’s release rate was studied for a period of ten weeks. Results confirmed that the films exhibited properties suitable for use in intraocular drug delivery systems with the single-layer thin films in particular demonstrating a sufficient encapsulation of dexamethasone and appropriate release of the therapeutic substance [34] while the IOLs were durable in spinning speeds higher than the ones used to develop thin films and their transparency and drug release rate was acceptable [32]. Tan et al. loaded a degradable poly(l-lactide-*co*-ε-caprolactone) polymer multilayer film with MFX and levofloxacin and attached it to a commercially available IOL and haptic [38]. Results indicated that levoflaxin would be released over a 14 day-period, appropriate for infection prevention.

There were two studies detailing mathematical models that assess the pharmacokinetic properties of drugs released by IOLs and backed by experimental data. Pimenta et al. [36] presented a mathematical model that aimed to estimate the concentration of drug released from an IOL in the eye aqueous humor. They validated the model by comparing the predicted aqueous humor concentrations with those obtained in MFX in vivo studies. They used the predicted drug concentrations in the aqueous humor to evaluate the best performing combination for preventing endophthalmitis, with the hydrophilic acrylic material demonstrating good results, particularly for diclofenac and MFX controlled-release. Gudnason et al. [30] introduced a general one-dimensional finite element drug delivery framework, which along with diffusion, reversible binding and dissolution within material layers, incorporated the partitioning and mass transfer conditions between layers of material. They compared their simulated MFX release data to experimental data and managed to explain three release curves, corresponding to different thicknesses of IOLs.

We have added in this part of the review a study by Topete et al. [39] on a very significant issue, that of researching appropriate sterilization methods for drug-loaded IOLs. The authors assessed the performance of high hydrostatic pressure (HHP) in the sterilization of a commercial acrylic material used for the production of IOLs, both without and loaded with an antibiotic (MFX) or an anti-inflammatory drug (ketorolac or diclofenac) and the results were compared to those obtained with the conventional sterilization methods of gamma radiation and steam heat. The authors reported considerable issues with the conventional methods since gamma radiation degraded the drugs while steam heat cannot be applied to temperature-sensitive drugs. However, HHP sterilized highly contaminated and also enhanced drug loading and did not affect significantly the hydrogel properties showing promise in addressing this significant hurdle.

Regarding potential risks and biases in the proposed methods, a general remark is that the authors have not commented on the associated costs, man-hours, or difficulty in achieving the presented outcomes. The success rate for the described IOL modifications in all instances is unclear, there is no mention of material failure during the modification process and no long-term assessment of shelf-life and ease-of-transport with the exception of the study by Vieira et al. [40] on shelf-life. Additionally, there are no direct comparison studies to other more well-established methods, save for the Topete et al. study [39]. The extent to which the new methods can be exploited commercially is thus unclear; although sterilization may not be an issue when novel methods are applied, as with HHP [39], the stability of drug-loaded IOLs in storage is in question; this is an important parameter since a drug-loaded IOL has to reach the surgeon without drug loss or discrepancies between the stated drug release profile and the actual one.

### 3.2. Studies That Examine the Therapeutic Impact of Specific Drugs in the Prevention of Adverse Outcomes Following IOL Implantation

The development of a modified IOL to act as a drug delivery device has been pursued for many years and is a promising concept in modern cataract surgery. There was considerable progress during the past five years in the follow-up studies to examine the therapeutic potential of specific drugs for the prevention of adverse outcomes following IOL implantation. These studies used the simpler method of soaking the IOL into a solution containing the examined substance. An overview of those seven studies is presented in Table 2.

Previously Kleinmann et al. [54] tested whether hydrophilic acrylate lenses could support the loading of the fluoroquinolones MFX and gatifloxacin. No adverse reactions were reported to those modified IOLs, while the sustained antibiotic release was achieved at minimum inhibitory concentrations to prevent endophthalmitis [54]. These results were recently replicated in vivo by Filipe et al. [47] by comparing two groups of animals that ether received topical MFX drops (control) or MFX-loaded IOLs. The IOLs provided a superior profile of controlled release of MFX for approximately 2 weeks without any differences in terms of inflammatory reactions, capsular bag opacification scores, and uveal and capsule biocompatibility. Since the Tan et al. study referenced above [38] conclude in a preference for levofloxacin versus MFX, it appears that so far MFX is better employed in the cruder soaking method. Two recent studies by Topete et al. examined the concomitant use of both MFX and the anti-inflammatory ketorolac in both hydrophilic and hydrophobic IOLs for the prevention of endopthalmitis [49] and PCO [50]. This reduced the tendency for adhesion of LECs and were able to release the medication at therapeutic levels in a sustained way while having better performance than ocular drops in the prevention of PCO.

A very promising class of active substances are the alkylphosphocholines, combining very good antiproliferative activity with good biocompatibility. It has been shown that this class of substances is particularly well tolerated by stem cells (bone marrow cells), with good effectiveness against proliferating cells at the same dose [55]. Erufosine has been deemed a suitable candidate for research on its applicability for IOL delivery since it is a potent inhibitor of phosphoinositide-3-kinase, an important molecular switch in post-cataract formation [56]. It has been loaded in IOLs and testing in a human capsular bag model found that it significantly delayed the development of cataracts over 45 days compared to controls [51].

Other active ingredients relevant for pharmacological post-cataract prophylaxis are inhibitors of the EGF receptor (EGF: epidermal growth factor) such as erlotinib [57] and gefitinib [58]. Both are promising in the preliminary studies so far. Two new and promising treatments for PCO targeting the epidermal growth factor receptor (EGFR) were presented in the studies by Wertheimer et al. [53] and Kassumeh et al. [48]. In the former study, the authors examined the potential of erlotinib, which is an inhibitor of the EGFR and reduces critical cellular events leading to PCO. They soaked IOLs with erlotinib and concluded that the modified IOLs mitigated cell growth in the anterior-segment model and the human ex vivo capsular bag model that they employed [59], without any relevant toxicity [53]. In the latter study, gefitinib, another EGFR inhibitor, was tested in a similar research design, with good effect since there was cell growth inhibition in non-toxic concentrations. The two EGFR inhibitors are not directly compared to each other.

Finally, a comparative study of a number of pharmacologic substances proposed for preventing PCO including caffeic acid phenethyl ester, disulfiram, methotrexate, rapamycin, and retinoic acid was carried out by Wertheimer et al. [52] in a similar setting as to the two studies by the same research team referenced above. Disulfiram was the only substance that failed to show an effect in the anterior segment model. Only methotrexate managed to demonstrate long-term inhibitory effects in the human capsular bag model, lack of toxicity and a constant release over two weeks.

A general remark following the review of these studies is that the field has progressed with well-designed, comparative studies; also, since all the drug compounds are not patented anymore, potential related biases are negated.

### 3.3. Alternatives to Medication against PCO and Infection

A number of alternative methods to protect against PCO and post-operative infections are becoming available; these methods overcome issues as the adhesion of cells and the increasing hurdle of growing antibacterial resistance among pathogens via specific coatings of the IOL with materials that either inhibit cell growth, bacterial adhesion or may even act bactericidally. A detailed list of those fifteen studies is presented in Table 3.

Farukhi et al. [61] expanded on earlier work by Matsushima et al. [75] who first demonstrated the feasibility of modifying the surface of IOLs by treating them with UV/O_3_ and argon plasma, resulting an increase in nitrogen substituents and –OH and –COOH functional groups. The –OH group is a main factor in improving hydrophilicity. The –COOH groups are highly adhesive to the adhesion protein fibronectin, thus increasing the adhesion of LECs and fibronectin and preventing PCO formation. In the new study the authors showed the superiority of UV/O_3_ modified IOLs compared to controls in a comparative study in rabbit eyes, biocompatibility was similar while the performance of an Nd:YAG laser posterior capsulotomy was not rendered more difficult by eventual adhesion between the posterior optic surface and the posterior capsule. On a similar note, Zheng et al. [74] tested various combinations of IOL amination by ammonia plasma and treatment with recombinant hirudin (rH) anticoagulant in vivo in rabbit eyes. Ammonia plasma introduces nitrogen substituents and also –OH and –COOH functional groups. The authors reported that the grafting of rH led to better resistance to cell adhesion than a pure amination process, while inhibiting the excessive proliferation of cells on the IOL.

Viveiros et al. [70] evaluated the adhesion of LECs on various hydrophilic acrylic intraocular lenses (IOLs) coated with polyethylene glycol (PEG) an in vitro study and found the coating effective in inhibiting cell adhesion. An in vivo study by Xu et al. [72] demonstrated effective prevention of PCO development in PEGylated IOLs since the initial adhesion of LECs was greatly inhibited while the process did not influence the lens optical properties.

Tan et al. [69] synthesized a hydrophilic copolymer (2-methacryloyloxyethyl phosphorylcholine-methacrylic acid) and grafted the copolymer onto the surface of IOL through air plasma treatment. The modified IOLs were demonstrating resistance to protein adsorption, and inhibition of adhesion and proliferation of lens epithelial cells in vitro. In vivo experiments confirmed significantly reduced postoperative inflammation and anterior capsule opacification (ACO), and did not affect PCO.

Syed Hussain et al. [68] examined the properties of zinc oxide nanocomposite polymer resins Films made with these poly(phenylethylacrylate-*co*-phenylethylmethacrylate)(poly(PEA-*co*-PEMA))oligomer-ZnO nanocomposites significantly resisted fibroblast attachment, filtered UV light and demonstrated appropriate intrinsic properties for their application in IOLs.

Mao et al. [67] assessed the properties of carboxylated CuInS/ZnS quantum dots (ZCIS QDs) and their potential as LEC-antiproliferative surface materials for IOL application, the end goal being a limited photothermal response after exposure to near-infrared radiation (NIR). The authors presented the fabrication process, results from characterization and an in vitro examination of photothermal properties of ZCIS QDs. They assessed biocompatibility in vitro with mouse fibroblast cells in a case-control design and concluded that combining QDs-IOLs and NIR irradiation achieves photothermal killing effects on LECs limited to the specific region with no cytotoxicity.

Choi et al. [60] described an ionic polymer-coated elastic nanopillar array (NPA) integrated onto an IOL, which demonstrated >99% antibacterial efficiency against *Staphylococcus aureus* by capturing and eradicating the bacteria through rapture of the bacterial membrane. The array was created by stamping a UV-curable polymeric blend of polyurethane acrylate (PU) and Norland Optical Adhesive 63 (NOA 63), called PUNO, on a prepatterned NPA mold. The authors claimed that the polymer coating and the specific NPA dimensions can decrease the adhesivity of corneal endothelial cells and posterior capsule opacification without causing cytotoxicity.

Wang et al. [71] covalently grafted a hyaluronic acid–lysozyme (HA–lysozyme) composite coating on the surface of PMMA intraocular lenses creating a highly hydrophilic surface with low roughness that lead to significantly reduced adherences of *Staphylococcus *aureus** and human LECs. There was also considerable bactericidal activity against *Staphylococcus *aureus** owing to the component of lysozyme.

Five years ago, Lin et al. managed to coat silicone-hydrogel IOLs with hyaluronic acid and chitosan polyelectrolyte multilayers to prevent PCO while preserving optimal optical qualities via the application of thin multilayers directly on IOLs [63]. The authors followed up on their work with a study of the antibacterial and anti-acute inflammatory properties of those compounds [64]. In vitro results showed that antibacterial activity is increasing with an increased number of layers while the modified IOLs demonstrated a marked reduction of inflammation compared to unmodified IOLs an in vivo controlled experiment. They further expanded on their work by modifying a commercially available IOL with silica coated Au nanorods (Au@SiO2) forming a photothermal ring which would react with the application of non-toxic NIR, eliminating the residual lens epithelial cells (LECs) around Nano-IOLs under mild laser treatment and block the formation of disordered LECs fibrosis, via its thermal effects. [65] The study included an in vivo case-control experiment with rabbits that confirmed good biocompatibility as well as very good effects with PCO occurrence in rabbit models at 30–40% at thirty days post-surgery, which was significantly lower than the control group that treated with C-IOLs (100% PCO occurrence). The authors concluded that the potential for spatial controllability of photothermal effect from nanomaterials to prevent PCO is considerable.

Zhang et al. [73] demonstrated the feasibility of polymerizing a novel bionic brushes coating onto the surface of an IOL that exhibited excellent antifouling efficiency against bovine serum albumin, *Staphylococcus aureus*, and human LECs, excellent antibacterial and antibiofilm abilities and good biocompatibility. The coating contained MPC and *N*,*N*,*N*-trimethyl-2-({4-[2-(4-nonylphenoxy)ethoxy]-4-oxobut-2-enoyl}oxy) ethan-1-aminium chloride (TOEAC), a quaternary ammonium monomer. It was prepared onto IOLs using reversible addition–fragmentation chain transfer polymerization method. From an in vivo experiment, the authors’ result confirmed that the coating effectively prevented PCO and endophthalmitis.

Magin et al. [66] determined the feasibility of employing sharklet-micropatterned protective membrane (PM) implanted in combination with a posterior chamber IOL to reduce chances of developing PCO. The authors carried out an in vitro assessment of several sharklet-styled microtopographies in a modified scratch-wound test on LEC migration. They found that all sharklet microtopographies significantly reduced LEC migration compared with a simple membrane and chose the best performing one for evaluation. This was compared to an un-patterned PM and an IOL without any PM. Results found that an IOL with a PM fitted with the best-performing micropatterned design had reduced LEC migration by 50% compared with the IOL-only condition. IOLs with simple PMs did not differ in LEC migration compared with the IOL only condition.

Seven out of the fifteen assessed studies had an in vivo well-designed case-control part; this points to the maturity of the field. However, as was the case with in Section 3.1., the authors have not commented on the associated costs, man-hours, or difficulty in achieving the presented outcomes. While comparisons as to the effectiveness are made to the controls with unmodified IOLs, the authors shy away from cost considerations. This detail may be critical in the era of managed care, where decisions will be made in comparison to less costly alternatives. We must of course concede that a researcher cannot adequately assess costs in a commercialized process and mass production does reduce expenditures. However, the authors could have provided data on the success rate for the described IOL modifications, material failure during the modification process and long-term assessment of shelf-life and ease-of-transport. The authors need to be more forthcoming with all relevant information in future studies.

### 3.4. Apparent Lack of Progress in Heparin IOL Surface Modification

While these developments appear promising, attention should also be given to cases of apparent lack of meaningful progress and this relates to heparin IOL surface modification. Although heparin-treated IOLs appear to provide benefits against inflammation [76], however, the case is not the same against PCO. Krall followed up on his earlier work on heparin-treated IOLs [76] with a randomized, double-blind, monocenter controlled clinical trial that sought to compare PCO rate at one year following implantation of a hydrophobic acrylic IOL with or without heparin coating [62]. They examined 102 eyes of 51 patients who underwent uneventful bilateral phacoemulsification with randomized IOL-type implantation. Results in both groups did not differ significantly on all measured parameters, and the authors rejected the initial postulation that decreased postoperative inflammatory reaction might have a beneficial effect on reducing PCO in long-term follow-up. Heparin in principle should reduce bacterial adhesion and the risk of infection while being well-tolerated. Yet, the clinical studies against infection and PCO are lacking.

### 3.5. Lack of Clinical Studies Currently Underway for a Commercially-Viable IOL Drug Delivery System

Following the apparent lack of a commercially available IOL drug delivery system we reviewed the WHO International Clinical Trials Registry Platform (ICTRP), ClinicalTrials.gov and the International Standard Randomized Controlled Trial Number (ISRCTN) registry for clinical trials in the field in order to identify the most promising systems, since systems selected for clinical trials would be strong candidates to enter a commercialization route.

The search in the ISRCTN and the ClinicalTrials.gov website included two keywords, IOL and cataract and it was carried out on clinical trials only; there were 382 and 344 trials identified respectively on the two registries. A single clinical study that was related ended in February 2008; it was a stage 2 clinical trial designed to test the effectiveness of an intraocular lens treated with heparin in reducing or preventing inflammation after cataract surgery in patients with uveitis (NCT00001311). There were 144 results in the smaller ISRCTN registry in the Eye Diseases category with the simple search term ‘cataract’; no related studies were recovered. We conclude that no results from a related clinical trial have been reported insofar while no clinical trials have been reported as being underway despite the fact that most original small-scale studies have been carried out some time ago and the original research teams have moved on to other fields.

### 3.6. Alternative Strategies for PCO Prevention

As of late, an alternative hypothesis for PCO has been proposed, stating that maintenance of at least some LECs is required to maintain the lens capsular bag [77]. This hypothesis directly relates to whether an IOL-based drug delivery system that kills all residual lens epithelial cells after cataract surgery might ultimately result in degradation of the lens capsule and consequently impaired vision due to axis rotation of toric IOLs or decentration of multifocal IOLs. Although two new studies have referenced this hypothesis [48,53], it has not featured in their design and methodology. Future clinical studies may have to take into account those results and aim for a limited inhibition of LEC proliferation.

A very promising concept for further research is partial lens regeneration after cataract surgery by taking advantage of the fibrotic growth that leads to PCO after cataract surgery and supplanting it with normal growth that leads to regeneration of lens structure and function. LECs provide a polarizing cue that is important for the alignment and orderly arrangement of lens fibers. Lovicu et al. suggested that the main requirement for this development would be to block TGF-β until the fiber differentiation response is underway, and added that this could be achieved by impregnating the IOL with a slow release form of an appropriate TGF-β blocking agent. This concept has not been put to the test yet in any of the reviewed studies.

### 3.7. Commercially Available Alternatives to IOL Drug Delivery

As mentioned before, there are no commercially available IOLs drug delivery systems. However, a number of simpler alternatives are gaining traction. “Dropless” cataract surgery is based on the intracameral injection of drugs during cataract surgery, in formulations specifically designed to provide prolonged drug release. The injections may be transzonular or intravitreal; two typical formulations are marketed by Imprimis Pharmaceuticals, Tri-Moxi (triamcinolone 15 mg/mL + 1 mg/mL MFX) and Tri-MoxiVanc (with added vancomycin). This procedure has been shown to be equally effective to eye-drops [78]. The patient needs to be alerted to the possibility of experiencing a brief reduction in visual acuity after surgery seeing and having a visual sensation of “floaters” due to the opaque nature of the combination of drugs. Interestingly, although both intracameral and IOL drug delivery systems have even been tested concurrently, showing an additive effect [79], this practice has not been repeated.

### 3.8. Overview of the Results and the Course of the Field

While new and promising methodologies for taking advantage of an implanted IOL as a carrier of active molecules or a matrix for appropriate surface modification have been presented, there is almost no progress towards a commercially available product to tackle the most important postoperative complications; inflammation, infection and PCO. Simpler methods to achieve the same stated goals however are gaining traction. It is unclear what the hurdles from transferring research results to practical application are. An ideal commercial product should have a manageable production cost, inexpensive and reliable sterilization, storage and transportation, a long shelf life and ease of use for the surgeon. Unfortunately, only two of the aforementioned studies attempted to address some of those very practical issues [39,40] and as a whole we do not have convincing answers to questions as to how shelf life may be affected by drug loading, or any special storage needs, etc. Although novel technologies as those described in the studies under Section 3.1 may be effective, their cost effectiveness remains to be seen.

### 3.9. Limitations of this Review

A major limitation of this review is that since no data synthesis is possible for the available data, due to considerable heterogeneity, we have only reported a qualitative analysis of the results and not a quantitative one. Furthermore, there is a possibility that the results from clinical trials that did not produce meaningful results were underreported; this would paint a misleading picture of more promise than in reality.

## 4. Materials and Methods

A limited review of the latest literature was conducted and reported in line with the criteria set out by the PRISMA guidelines [26]. Eligibility criteria were: original pre-clinical or clinical studies with an experimental part, presented in English, during the past five years, on IOL drug delivery systems and surface modifications associated with the prevention or treatment of postoperative complications of cataract surgery. MEDLINE (accessed from PubMed) from 1 January 2015 to 22 October 2020, Scopus and Web of Science (WoS) were systematically searched for related published articles. A review protocol was not previously submitted. The rationale for the timeframe was chosen to best provide with the latest advances in the field. The choice of MEDLINE was made to focus on applied science rather than basic science.

The NLM/PubMED and WoS search string is: “((Intraocular lens*) OR (IOL)) AND ((drug*) AND ((deliver*) OR (releas*)) OR ((surface modif*) OR (coat*))) NOT (glaucoma) NOT (contact) NOT (labor)” during the past five years; the terms that were specified not to be included related to instances of describing glaucoma treatment, contact lenses and obstetrics papers since the IOL term is common with obstetrics where it stands for induction of labor. The term ‘cataract’ was not included on the final search string as it was found to be too restrictive. Both authors systematically screened the search results independently and applied the inclusion and exclusion criteria.

## 5. Conclusions

The significant potential for the use of IOLs as drug delivery reservoirs to prevent infection, inflammation and PCO following cataract surgery has been researched for more than twenty-five years now. While considerable progress is continually being made with regard to methods and materials, there is still little capitalization upon these research studies, with no commercially available IOL-based drug delivery system being available. This delay has opened the door to less complicated alternatives, such as the intracameral injection of drug compounds; hence the field of potential use may be narrowing. However, modification of the surface of the IOLs with novel materials that exhibit potent antibacterial effect and/or present a considerable obstacle to the proliferation of LECs is a field of application unique to this route of administration and thus of significant clinical interest. Unfortunately, we still remain in need of additional clinical data in order to apply these advances to the treatment of patients, much as we were before some time [80]. Close cooperation between researchers in basic sciences (chemistry, physics, materials science and pharmacy), clinical researchers, IOL manufacturers and the pharmaceutical industry is an important prerequisite for further development.

## Figures and Tables

**Table 1:** Included studies relating to new methods for IOL loading with active molecules over the past years.

Authors	Type of Study	Aim	Procedure	Outcome
Artigas et al. [27]	In vitro, three-way comparison study	Evaluation of dexamethasone-loaded IOL’s optical properties	Dexamethasone loading by soaking the IOL in a polymer matrix	Drug loading impairs modulation transfer function and spectral transmission; they recover after the drug is released
Bouledjouidja et al. [28]	In vitro single-group experimental study	Development of supercritical impregnation for IOL drug loading	IOL supercritical impregnation with ciprofloxacin (CIP) and dexamethasone 21-phosphate disodium (DXP)	Higher affinity for DXP compared to CIP, highest DXP impregnation yields were obtained in the presence of ethanol as a co-solvent, unlike with CIP
Bouledjouidja et al. [29]	In vitro single-group experimental study	Examination of optical properties in IOLs loaded with supercritical impregnation	IOL supercritical impregnation with ciprofloxacin (CIP) and dexamethasone 21-phosphate disodium (DXP)	Supercritical impregnation does not damage the optical properties of IOLs
Gudnason et al. [30]	In vitro comparison of simulated and experimental data	Evaluation of a mathematical model to estimate the concentration of drug released from drug-loaded IOLs via in vitro calculations verified against in vivo measurements	The authors compared their simulated moxifloxacin (MFX) release data to experimental data from MFX-loaded IOLs	The authors managed to explain three release curves, corresponding to different thicknesses of IOLs.
Han et al. [31]	In vitro observations and in vivo single-group experimental study	Manufacture and evaluation of an antiproliferative drug: doxorubicin (DOX)-incorporated chitosan (CHI) nanoparticle on cellular adhesion, proliferation and migration, in vivo evaluation of PCO inhibitory effect	Drug-loaded multilayer fabrication and coating onto IOL by multi-stage injection, in vivo cell migration experiment, in vivo PCO inhibition in rabbit eyes for up to two months	In vitro cell adhesion was reduced, cell migration and proliferation were remarkably inhibited and PCO formation after drug-eluting IOL implantation was significantly inhibited.
Karamitsos et al. [32]	In vitro observational study	Design, development and study of the properties of a novel polymeric, drug-eluting thin film and its application on an IOL with dexamethasone (DXM)	Examination of the initial durability of the IOLs during the spinning process and of structural and optical properties of the modified IOLs. A drug release study run for 8 weeks	There was acceptable optical transparency of the lenses regardless of the deposition of the drug-eluting films on their surface. The drug release study demonstrated gradual DXM release over the selected period.
Kassumeh et al. [33]	In vitro and ex-vivo experimental three-group study	Determine the feasibility of methotrexate (MTX) loaded biomatrices sprayed on IOLs	Evaluation for IOL growth-inhibiting properties, release kinetics of MTX and its toxicity on corneal endothelial cells	Significant benefits compared to controls on all parameters with no difference in toxicity
Lamprogiannis et al. [34]	In vitro observational study	Design, development, characterization, and drug release of one- and two-layered thin films based on organic polymers and DXM	Examination of the opacity and rate of DXM release over a six-week period.	The single-layer thin films demonstrated a sufficient encapsulation of dexamethasone and appropriate release of the therapeutic substance over a six-week period.
Ongkasin et al. [35]	In vitro observations and ex vivo case-control study	Application of supercritical impregnation technology to load acrylic IOLs with MTX to produce a sustained drug delivery device to mitigate PCO	In vitro assessment of drug release kinetic employed to appropriately load the IOLs. Measurement of time to full coverage of the optical axis with PCO cells	No statistically significant variation in the duration required for a full cell coverage of the posterior capsule ex vivo. Reduction in fibrosis by inhibiting epithelial-mesenchymal transformation.
Pimenta et al. [36]	In vitro observations and in vivo multiple-group experimental study	Evaluation of a mathematical model to estimate the concentration of drug released from drug-loaded IOLs via in vitro calculations verified with in vivo experiments	Partition and effective diffusivity values were determined for MFX, levofloxacin, diclofenac and ketorolac in hydrophilic acrylic IOLs and in silicone hydrogel.	The hydrophilic acrylic material presented promising results, especially for MFX and diclofenac controlled-release
Sahariah et al. [37]	In vitro experimental study	Manufacture and evaluation of three chitosan derivatives for suitability as a drug carrier for MFX	Photografting and photocrosslinking of three polymethacrylate copolymers and in vitro comparison of drug release profiles	The authors reported an optimized procedure for synthesizing chitosan derivatives of high molecular weight and determined the best performing derivative in terms of highest amount of released drug.
Tan et al. [38]	In vitro experimental study	Evaluation of loading IOLs with a fully-degradable polymer depot with levofloxacin or MFX against infection.	The effects of drug loading and solvent type on drug release and film morphology were investigated using cast films.	A slower-evaporating solvent tetrahydrofuran and a lower drug loading percentage led to better surface morphology and lower initial release burst compared to dichloromethane and higher drug loading percentages.
Topete et al. [39]	In vitro case-control study	Assessment of sterilization of a drug-loaded IOL by high hydrostatic pressure (HHP)	Comparative study of the effectiveness of HHP against gamma radiation and steam heat on IOLs loaded with MFX or an anti-inflammatory drug	Gamma radiation degraded the drugs while steam heat cannot be applied to temperature-sensitive drugs. However, HHP sterilized highly contaminated and also enhanced drug loading and did not affect significantly the hydrogel properties.
Vieira et al. [40]	In vitro observational study	Manufacture and evaluation of the effects and properties of MFX-loading on pHEMA-silicone IOLs	Characterization of MFX-loaded IOLs and study of antibacterial, cytotoxic and storage properties	The MFX-loaded IOLs were effective against *S. aureus and S. epidermidis* without cytotoxicity. While there was no case of drug loss during three months of storage, an increase in storage time lead to an increase in the amount of MFX released and to an increase in its release duration

**Table 2:** Included studies of the effectiveness of specific drugs for IOL loading over the past five years.

Authors	Type of Study	Aim	Procedure	Outcome
Filipe et al. [47]	In vitro observations and in vivo case-control experimental study	To assess IOLs loaded with moxifloxacin (MFX) for drug release activity, cytotoxicity and efficacy against bacterial infection.	The activity of the released drug was tested in vitro while in vivo cytotoxicity and efficacy was evaluated comparing the effects of topical MFX drops (control) and MFX-loaded IOLs	The presence of MFX in the IOLs had little effect on the evaluated physical properties and did not induce cytotoxicity. In vitro drug release experiments showed that the IOLs provided controlled release of MFX for ~2 weeks with less variability than controls.
Kassumeh et al. [48]	In vitro observations and ex vivo case-control study	Evaluation of loading IOLs with gefitinib against the development of PCO.	In vitro observations of rates of LEC growth in an anterior segment model, followed by in vivo experiment to determine drug release and biocompatibility in a human capsular bag model	Coated IOLS attenuated PCO cell growth with a constant drug release over the first ten days, without any reduction in cell viability of corneal endothelial cells and with a related decrease in fibronectin expression.
Topete et al. [49]	In vitro multiple-group observational study	To assess the possibility of loading IOLs with a combination of an antibiotic and an anti-inflammatory drug, either MFX + ketorolac (KTL) or MFX + diclofenac	Drug-loaded IOLs were assessed for their optical and mechanical properties and the time duration of effective dosing was calculated	Simultaneous drug loading improved the release profiles with no adverse impact on the optical and mechanical properties. The most effective combination was a loading with MFX + KTL, effective against *S. aureus* and *S. epidermidis* for up to 15 days and against inflammation for at least 16 days.
Topete et al. [50]	In vitro observations and in vivo case-control experimental study	Evaluation of loading IOLs with MFX + KTL against the development of infection and inflammation.	In vitro drug release tests and the antimicrobial activity of the released antibiotic was determined while the in vivo performance and safety of both hydrophobic and hydrophilic lenses was compared to that of ophthalmic drops.	The developed IOLs were able to release MFX and KTL at therapeutic levels, in a sustained way, unlike eye drops prophylaxis. No PCO signs were detected and histological analyses demonstrated biocompatibility of these devices.
Wertheimer et al. [51]	Ex vivo and in vitro case-control experimental study	Evaluation of whether erufosine alone or erufosine-loaded IOLs can inhibit growth of LEC	Tissue from cadaver eyes that underwent sham surgery was exposed to erufosine while IOLs soaked with erufosine were implanted in capsular bags	Erufosine as a single therapeutic agent increased the time until confluence of the capsular bag, but not significantly compared with the control. When IOLs were soaked with erufosine, a long-term prophylactic effect was observed in this organ model for PCO
Wertheimer et al. [52]	Ex vivo multi-group comparative study following in vitro study	Evaluation of various substances for loading on IOLs for PCO prevention	In vitro screening of potential candidate substances for toxicity and testing for effect on PCO with IOLs in vitro and ex vivo	Long-term inhibitory effects in the human capsular bag model were observed for caffeic acid phenethyl ester and methotrexate IOLs. Only methotrexate and disulfiram were not toxic. Methotrexate was released constantly for two weeks
Wertheimer et al. [53]	Ex vivo and in vitro case-control experimental study	Evaluation of whether erlotinib-loaded IOLs can prevent PCO while not being toxic	Tissue from cadaver eyes that underwent sham surgery was exposed to erlotinib while IOLs soaked with erlotinib were implanted in capsular bags	Modified IOLs mitigated cell growth in the anterior segment model without short-term corneal toxicity while there was no toxicity on corneal endothelial cells. Erlotinib was released constantly from IOL.

**Table 3:** Included studies examining non-pharmaceutical IOL surface modifications over the past five years.

Authors	Type of Study	Aim	Procedure	Outcome
Choi et al. [60]	In vitro experimental study	To assess the feasibility of a integrating a polymeric nanopillar array (NPA) onto an IOL to prevent infection, decrease the adhesivity of LEC and prevent PCO without cytotoxicity	In vitro comparison of an NPA coated with cross-linked ionic polymer thin film (pVD) to a bare NPA as to the anti-bacterial efficiency.	The pVD-coated NPA exhibited excellent anti-bacterial efficiency >99.6%. The bare NPA only showed 51% efficiency stemming from the topological bacteria-killing effect
Farukhi et al. [61]	In vivo case-control experimental study	To assess the effectiveness of treating an IOL with ultraviolet–ozone (UV–O_3_) on the posterior surface for prevention of PCO	The UV–O_3_ treated IOL was compared for effectiveness to an identical untreated IOL in a rabbit model	Treatment of an IOL with UV–O_3_ appears to prevent PCO with no signs of untoward inflammation or toxicity or any other difference in histopathologic findings between study eyes and control eyes.
Krall et al. [62]	In vivo case-control human clinical study	To assess the performance of a heparin surface modified IOL (HSM-IOL) for benefit against PCO to an uncoated IOL (UC-IOL) 1 year after implantation.	The heparin surface modified IOL was compared for effectiveness to an identical untreated IOL in human patients	Patients were compared in PCO grading, straylight measurement, distance visual acuities, flare in the anterior chamber, and mesopic and photopic contrast sensitivity. There were no statistically significant differences between the groups.
Lin et al. [63]	In vitro observations and in vivo single-group experimental study	Design, development and study of the properties of a novel hyaluronic acid (HA)/chitosan (CHI) polyelectrolyte multilayer for IOL application	In vitro observations of protein adsorption and LEC adhesion/ proliferation followed by in vivo experiment after IOL implantation	In vitro results showed no reduction of the optical properties and inhibition of LEC adhesion and proliferation while in vivo ocular implantation results showed good biocompatibility and reduction of PCO.
Lin et al. [64]	In vitro observations and in vivo single-group experimental study	Further research of a previously presented HA/CHI polyelectrolyte multilayer for antibacterial and anti-acute inflammatory properties	In vitro antibacterial adhesion test followed by in vivo experiment after IOL implantation	In vitro results showed that antibacterial activity is increasing with an increased number of layers while the modified IOLs demonstrated a marked reduction of inflammation compared to unmodified IOLs
Lin et al. [65]	In vitro observations and in vivo case-control experimental study	To assess the feasibility of grafting a nanostructure photothermal ring onto an IOL (nano-IOL) to prevent infection, decrease the adhesivity of LEC and prevent PCO without cytotoxicity	In vitro examination of the properties of the components of the nanostructure and of the nano-IOL. The nano-IOL was compared in vivo to a regular IOL as to LEC proliferation	The nano-IOL demonstrated good biocompatibility, region-confined photothermal effect and no toxicity. PCO occurrence after surgery was 30% in eyes with nano-IOLs compared to 100% of eyes with untreated IOLs
Magin et al. [66]	In vitro observations and controlled study	To determine the feasibility of employing sharklet-micropatterned protective membrane (PM) implanted in combination with a posterior chamber IOL to improve resistance to PCO.	In vitro assessment of several microtopographies in a modified scratch-wound test to assess LEC migration, best performing was compared to an un-patterned PM and an IOL without any PM	An IOL with a PM fitted with the best-performing micropatterned design had reduced LEC migration by 50% compared with the IOL-only condition. IOLs with simple PMs did not differ in LEC migration compared with the IOL only condition.
Mao et al. [67]	In vitro observational and case-control group study	To assess the properties of carboxylated CuInS/ZnS quantum dots (ZCIS QDs) and their potential as LEC-antiproliferative surface materials to IOLs	Fabrication, characterization and in vitro examination of photothermal properties of ZCIS QDs. In vitro case-control examination of biocompatibility	The results indicated that combining QDs-IOLs and NIR irradiation achieves photothermal killing effects on LECs limited to the specific region with no cytotoxicity.
Syed Hussain et al. [68]	In vitro observational study	To assess the properties of zinc oxide nanocomposite resins and their potential as LEC-antiproliferative surface materials to IOLs	Films made with the resins underwent an in vitro cytotoxicity test, a morphological study of attached cells and a fibroblast adherence assay.	An extensive presentation of the films’ properties concluded that they resisted fibroblast attachment, filtered harmful UV light and had appropriate visible light transparency, glass transition temperatures, mechanical strength, and biocompatibility
Tan et al. [69]	In vitro observations and in vivo case-control experimental study	To assess the feasibility of grafting a hydrophilic copolymer P(MPC-MAA) onto an IOL to reduce inflammation, decrease the adhesivity of LEC and prevent ACO and PCO without cytotoxicity	The assessment of the characteristics of the copolymer P(MPC-MAA) in vitro was followed by an in vivo examination in a case-control experimental design	P(MPC-MAA) modification significantly reduced postoperative inflammation and ACO, but did not affect PCO.
Viveiros et al. [70]	Ex vivo case-control experimental study	To evaluate the adhesion of LEC on IOLs coated with polyethylene glycol (PEG).	An ex vivo case-control experimental design to compare rate of adhesion for LECs	PEG-coated IOLs was effective in inhibiting cell adhesion
Wang et al. [71]	In vitro observational study	To assess the feasibility of grafting a Hyaluronic acid–lysozyme (HA–lysozyme) composite coating onto an IOL to decrease the adhesivity of LEC and *S. aureus* and prevent PCO	HA-IOLs and HA-lysozyme IOLs underwent surface characterization, cytotoxicity assays and an antibacterial test	Adherence of *S. aureus* and LECs on IOLs with HA or HA–lysozyme coating was significantly reduced while the bactericidal activity of HA–lysozyme coatings was effective against *S. aureus*
Xu et al. [72]	In vitro observations and in vivo case-control experimental study	To assess the performance of hydrophilic polyethylene glycol (PEG) as an IOL surface graft to inhibit LEC adhesion.	In vitro examination of the properties of the PEGylated IOLs that were subsequently compared to plain IOLs in an in vivo case-control study	PEGylated IOLs retained their optical properties with inhibited LEC initial adhesion. They presented good in vivo biocompatibility, and effective prevention of PCO.
Zhang et al. [73]	In vitro observations and in vivo case-control experimental study	To assess the feasibility of grafting a bionic zwitterionic polymer onto an IOL to resist nonspecific proteins, bacterial and LEC adhesion	In vitro examination of the properties of the polymer and description of the forming of P(TOEAC-*co*-MPC) brushes. The brushes were prepared onto IOLs and compared to plain IOLs	The P(TOEAC-*co*-MPC) brushes showed excellent antibacterial and antibiofilm abilities, good biocompatibility while the in vivo study confirmed that they effectively prevented PCO and endophthalmitis
Zheng et al. [74]	In vitro observations and in vivo case-control experimental study	To assess the feasibility of grafting a hirudin polymer onto an IOL to resist inflammation, bacterial and LEC adhesion	In vitro examination of the properties of the polymer. Hirudin-IOLs were compared to plain IOLs in an in vivo case-control study	Grafting hirudin to the IOL surface led to better resistance to cell adhesion than a pure amination process and reduced in vivo the incidence of cell aggregation and inflammation.

## References

[1] Addo E., Bamiro O.A., Siwale R. (2016). Anatomy of the eye and common diseases affecting the eye. Ocular Drug Delivery: Advances, Challenges and Applications.

[2] Kanski J.J., Bowling B. (2015). Clinical Ophthalmology: A Systematic Approach.

[3] Solebo A.L., Rahi J.S. (2017). Epidemiology of congenital cataract. Congenital Cataract.

[4] Morrison D.G., Umfress A.C. (2020). Secondary and acquired cataracts. Pediatric Cataract Surgery and Iol Implantation.

[5] Davis G. (2016). The evolution of cataract surgery. Mo. Med..

[6] Apple D.J., Sims J. (1996). Harold ridley and the invention of the intraocular lens. Surv. Ophthalmol..

[7] Tetz M., Jorgensen M.R. (2015). New hydrophobic iol materials and understanding the science of glistenings. Curr. Eye Res..

[8] Toro M.D., Nowakowska D., Brzozowska A., Reibaldi M., Avitabile T., Bucolo C., Murabito P., Chisari C., Nowomiejska K., Rejdak R. (2020). Pain following the use of anesthesia formulation among individuals undergoing cataract surgery: A randomized controlled trial. Front. Pharmacol..

[9] Mylona I., Dermenoudi M., Glynatsis M., Ziakas N., Tsinopoulos I. (2020). Development of a reliable preoperative risk stratification system for phacoemulsification. J. Cataract Refract. Surg..

[10] Toro M.D., Longo A., Avitabile T., Nowomiejska K., Gagliano C., Tripodi S., Choragiewicz T., Kaminska A., Figus M., Posarelli C. (2019). Five-year follow-up of secondary iris-claw intraocular lens implantation for the treatment of aphakia: Anterior chamber versus retropupillary implantation. PLoS ONE.

[11] Wormstone I.M., Wormstone Y.M., Smith A.J.O., Eldred J.A. (2020). Posterior capsule opacification: What’s in the bag?. Prog. Retin. Eye Res..

[12] Zhao Y., Yang K., Li J., Huang Y., Zhu S. (2017). Comparison of hydrophobic and hydrophilic intraocular lens in preventing posterior capsule opacification after cataract surgery: An updated meta-analysis. Medicine (Baltimore).

[13] Schaumberg D.A., Dana M.R., Christen W.G., Glynn R.J. (1998). A systematic overview of the incidence of posterior capsule opacification. Ophthalmology.

[14] Wesolosky J.D., Tennant M., Rudnisky C.J. (2017). Rate of retinal tear and detachment after neodymium: Yag capsulotomy. J. Cataract Refract. Surg..

[15] Knight P.M., Link W.J. (1979). Surface modification of intraocular lenses to reduce corneal endothelial damage. J. Am. Intra-Ocul. Implant.

[16] Nishi O., Nishi K., Yamada Y., Mizumoto Y. (1995). Effect of indomethacin-coated posterior chamber intraocular lenses on postoperative inflammation and posterior capsule opacification. J. Cataract Refract. Surg..

[17] Tetz M.R., Ries M.W., Lucas C., Stricker H., Völcker H. (1996). Inhibition of posterior capsule opacification by an intraocular-lens-bound sustained drug delivery system: An experimental animal study and literature review. J. Cataract Refract. Surg..

[18] An J.A., Kasner O., Samek D.A., Lévesque V. (2014). Evaluation of eyedrop administration by inexperienced patients after cataract surgery. J. Cataract Refract. Surg..

[19] Helary G., Yammine P., Migonney V. (2004). Surface modification of hydrogel intraocular lenses to prevent cell proliferation. J. Appl. Biomater. Biomech..

[20] Bozukova D., Pagnoulle C., De Pauw-Gillet M.-C., Desbief S., Lazzaroni R., Ruth N., Jérôme R., Jérôme C. (2007). Improved performances of intraocular lenses by poly (ethylene glycol) chemical coatings. Biomacromolecules.

[21] Kirchhof S., Goepferich A.M., Brandl F.P. (2015). Hydrogels in ophthalmic applications. Eur. J. Pharm. Biopharm..

[22] González-Chomón C., Concheiro A., Alvarez-Lorenzo C. (2011). Drug-eluting intraocular lenses. Materials.

[23] Verma L., Chakravarti A. (2017). Prevention and management of postoperative endophthalmitis: A case-based approach. Indian J. Ophthalmol..

[24] Chirila T., Harkin D. (2016). Biomaterials and Regenerative Medicine in Ophthalmology.

[25] Siqueira R.C., Ribeiro Filho E., Fialho S.L., Lucena L.R., Maia Filho A., Haddad A., Jorge R., Scott I.U., da Silva Cunha A. (2006). Pharmacokinetic and toxicity investigations of a new intraocular lens with a dexamethasone drug delivery system: A pilot study. Ophthalmologica.

[26] Moher D., Liberati A., Tetzlaff J., Altman D.G., Group P. (2009). Preferred reporting items for systematic reviews and meta-analyses: The prisma statement. PLoS Med..

[27] Artigas J.M., García-Domene M.C., Navea A., Botella P., Fernández E. (2017). Intra-ocular lens optical changes resulting from the loading of dexamethasone. Biomed. Opt. Express.

[28] Bouledjouidja A., Masmoudi Y., Sergent M., Trivedi V., Meniai A., Badens E. (2016). Drug loading of foldable commercial intraocular lenses using supercritical impregnation. Int. J. Pharm..

[29] Bouledjouidja A., Masmoudi Y., Li Y., He W., Badens E. (2017). Supercritical impregnation and optical characterization of loaded foldable intraocular lenses using supercritical fluids. J. Cataract Refract. Surg..

[30] Gudnason K., Sigurdsson S., Snorradottir B.S., Masson M., Jonsdottir F. (2018). A numerical framework for drug transport in a multi-layer system with discontinuous interlayer condition. Math. Biosci..

[31] Han Y., Tang J., Xia J., Wang R., Qin C., Liu S., Zhao X., Chen H., Lin Q. (2019). Anti-adhesive and antiproliferative synergistic surface modification of intraocular lens for reduced posterior capsular opacification. Int. J. Nanomed..

[32] Karamitsos A., Lamprogiannis L., Karagkiozaki V., Laskarakis A., Papadopoulou L., Fatouros D., Ziakas N., Logothetidis S., Tsinopoulos I. (2020). Design, characterisation and drug release study of polymeric, drug-eluting single layer thin films on the surface of intraocular lenses. IET Nanobiotechnol..

[33] Kassumeh S.A., Wertheimer C.M., von Studnitz A., Hillenmayer A., Priglinger C., Wolf A., Mayer W.J., Teupser D., Holdt L.M., Priglinger S.G. (2018). Poly(lactic-co-glycolic) acid as a slow-release drug-carrying matrix for methotrexate coated onto intraocular lenses to conquer posterior capsule opacification. Curr. Eye Res..

[34] Lamprogiannis L., Karamitsos A., Karagkiozaki V., Tsinopoulos I., Gioti M., Fatouros D.G., Dimitrakos S., Logothetidis S. (2018). Design and fabrication of drug-eluting polymeric thin films for applications in ophthalmology. IET Nanobiotechnol..

[35] Ongkasin K., Masmoudi Y., Wertheimer C.M., Hillenmayer A., Eibl-Lindner K.H., Badens E. (2020). Supercritical fluid technology for the development of innovative ophthalmic medical devices: Drug loaded intraocular lenses to mitigate posterior capsule opacification. Eur. J. Pharm. Biopharm..

[36] Pimenta A.F.R., Serro A.P., Colaço R., Chauhan A. (2018). Drug delivery to the eye anterior chamber by intraocular lenses: An in vivo concentration estimation model. Eur. J. Pharm. Biopharm..

[37] Sahariah P., Vieira A.P., Guiomar A.J., Alves P., Másson M. (2020). Utilization of tbdms chitosan for synthesis of photoactive chitosan derivatives and application in photografting on ophthalmic lens material. React. Funct. Polym..

[38] Tan D.W., Lim S.G., Wong T.T., Venkatraman S.S. (2016). Sustained antibiotic-eluting intra-ocular lenses: A new approach. PLoS ONE.

[39] Topete A., Pinto C.A., Barroso M.H., Saraiva J.A., Barahona I., Saramago B., Serro A.P. (2020). High hydrostatic pressure (hhp) as sterilization method for drug-loaded intraocular lenses. ACS Biomater. Sci. Eng..

[40] Vieira A.P., Pimenta A.F., Silva D., Gil M.H., Alves P., Coimbra P., Mata J.L., Bozukova D., Correia T.R., Correia I.J. (2017). Surface modification of an intraocular lens material by plasma-assisted grafting with 2-hydroxyethyl methacrylate (hema), for controlled release of moxifloxacin. Eur. J. Pharm. Biopharm..

[41] Zhang L., Zheng G.-J., Guo Y.-T., Zhou L., Du J., He H. (2014). Preparation of novel biodegradable phema hydrogel for a tissue engineering scaffold by microwave-assisted polymerization. Asian Pac. J. Trop. Dis..

[42] Garty S., Shirakawa R., Warsen A., Anderson E.M., Noble M.L., Bryers J.D., Ratner B.D., Shen T.T. (2011). Sustained antibiotic release from an intraocular lens–hydrogel assembly for cataract surgery. Investig. Ophthalmol. Vis. Sci..

[43] Anderson E.M., Noble M.L., Garty S., Ma H., Bryers J.D., Shen T.T., Ratner B.D. (2009). Sustained release of antibiotic from poly (2-hydroxyethyl methacrylate) to prevent blinding infections after cataract surgery. Biomaterials.

[44] Duarte A.R.C., Simplicio A.L., Vega-Gonzalez A., Subra-Paternault P., Coimbra P., Gil M., de Sousa H.C., Duarte C.M. (2008). Impregnation of an intraocular lens for ophthalmic drug delivery. Curr. Drug Deliv..

[45] González-Chomón C., Braga M.E., de Sousa H.C., Concheiro A., Alvarez-Lorenzo C. (2012). Antifouling foldable acrylic iols loaded with norfloxacin by aqueous soaking and by supercritical carbon dioxide technology. Eur. J. Pharm. Biopharm..

[46] Han Y., Xu X., Wang Y., Liu S., Zhao X., Chen H., Lin Q. (2018). Drug eluting intraocular lens surface modification for pco prevention. Colloids Interface Sci. Commun..

[47] Filipe H.P., Bozukova D., Pimenta A., Vieira A.P., Oliveira A.S., Galante R., Topete A., Masson M., Alves P., Coimbra P. (2019). Moxifloxacin-loaded acrylic intraocular lenses: In vitro and in vivo performance. J. Cataract Refract. Surg..

[48] Kassumeh S., Kueres A., Hillenmayer A., von Studnitz A., Elhardt C., Ohlmann A., Priglinger S.G., Wertheimer C.M. (2019). Development of a drug-eluting intraocular lens to deliver epidermal growth factor receptor inhibitor gefitinib for posterior capsule opacification prophylaxis. Eur. J. Ophthalmol..

[49] Topete A., Serro A., Saramago B. (2019). Dual drug delivery from intraocular lens material for prophylaxis of endophthalmitis in cataract surgery. Int. J. Pharm..

[50] Topete A., Tang J., Ding X., Filipe H.P., Saraiva J.A., Serro A.P., Lin Q., Saramago B. (2020). Dual drug delivery from hydrophobic and hydrophilic intraocular lenses: In-vitro and in-vivo studies. J. Control. Release.

[51] Wertheimer C., Brandlhuber U., Kook D., Mayer W.J., Laubichler P., Wolf A., Kampik A., Eibl-Lindner K. (2015). Erufosine, a phosphoinositide-3-kinase inhibitor, to mitigate posterior capsule opacification in the human capsular bag model. J. Cataract Refract. Surg..

[52] Wertheimer C., Kassumeh S., Piravej N.P., Nilmayer O., Braun C., Priglinger C., Luft N., Wolf A., Mayer W.J., Priglinger S.G. (2017). The intraocular lens as a drug delivery device: In vitro screening of pharmacologic substances for the prophylaxis of posterior capsule opacification. Investig. Ophthalmol. Vis. Sci..

[53] Wertheimer C., Kueres A., Siedlecki J., Braun C., Kassumeh S., Wolf A., Mayer W., Priglinger C., Priglinger S., Eibl-Lindner K. (2018). The intraocular lens as a drug delivery device for an epidermal growth factor–receptor inhibitor for prophylaxis of posterior capsule opacification. Acta Ophthalmol..

[54] Kleinmann G., Apple D.J., Chew J., Hunter B., Stevens S., Larson S., Mamalis N., Olson R.J. (2006). Hydrophilic acrylic intraocular lens as a drug-delivery system for fourth-generation fluoroquinolones. J. Cataract Refract. Surg..

[55] Dawes L.J., Illingworth C.D., Wormstone I.M. (2012). A fully human in vitro capsular bag model to permit intraocular lens evaluation. Investig. Ophthalmol. Vis. Sci..

[56] Liegl R., Wertheimer C., Kernt M., Docheva D., Kampik A., Eibl-Lindner K.H. (2014). Attenuation of human lens epithelial cell spreading, migration and contraction via downregulation of the pi3k/akt pathway. Graefes Arch. Clin. Exp. Ophthalmol..

[57] Wertheimer C., Liegl R., Kernt M., Mayer W., Docheva D., Kampik A., Eibl-Lindner K.H. (2013). Egf receptor inhibitor erlotinib as a potential pharmacological prophylaxis for posterior capsule opacification. Graefes Arch. Clin. Exp. Ophthalmol..

[58] Wertheimer C., Siedlecki J., Kook D., Mayer W.J., Wolf A., Klingenstein A., Kampik A., Eibl-Lindner K. (2015). Egfr inhibitor gefitinib attenuates posterior capsule opacification in vitro and in the ex vivo human capsular bag model. Graefes Arch. Clin. Exp. Ophthalmol..

[59] Gotoh N., Perdue N.R., Matsushima H., Sage E.H., Yan Q., Clark J.I. (2007). An in vitro model of posterior capsular opacity: Sparc and tgf-β2 minimize epithelial-to-mesenchymal transition in lens epithelium. Investig. Ophthalmol. Vis. Sci..

[60] Choi G., Song Y., Lim H., Lee S.H., Lee H.K., Lee E., Choi B.G., Lee J.J., Im S.G., Lee K.G. (2020). Antibacterial nanopillar array for an implantable intraocular lens. Adv. Healthc. Mater..

[61] Farukhi M.A., Werner L., Kohl J.C., Gardiner G.L., Ford J.R., Cole S.C., Vasavada S.A., Noristani R., Mamalis N. (2015). Evaluation of uveal and capsule biocompatibility of a single-piece hydrophobic acrylic intraocular lens with ultraviolet-ozone treatment on the posterior surface. J. Cataract Refract. Surg..

[62] Krall E.M., Arlt E.M., Jell G., Strohmaier C., Moussa S., Dexl A.K. (2015). Prospective randomized intraindividual comparison of posterior capsule opacification after implantation of an iol with and without heparin surface modification. J. Refract. Surg..

[63] Lin Q., Xu X., Wang B., Shen C., Tang J., Han Y., Chen H. (2015). Hydrated polysaccharide multilayer as an intraocular lens surface coating for biocompatibility improvements. J. Mater. Chem. B.

[64] Lin Q.K., Xu X., Wang Y., Wang B., Chen H. (2017). Antiadhesive and antibacterial polysaccharide multilayer as iol coating for prevention of postoperative infectious endophthalmitis. Int. J. Polym. Mater..

[65] Lin Y.-X., Hu X.-F., Zhao Y., Gao Y.-J., Yang C., Qiao S.-L., Wang Y., Yang P.-P., Yan J., Sui X.-C. (2017). Photothermal ring integrated intraocular lens for high-efficient eye disease treatment. Adv. Mater..

[66] Magin C.M., May R.M., Drinker M.C., Cuevas K.H., Brennan A.B., Reddy S.T. (2015). Micropatterned protective membranes inhibit lens epithelial cell migration in posterior capsule opacification model. Transl. Vis. Sci. Technol..

[67] Mao Y., Yu S., Kang Y., Zhang D., Wu S., Zhang J., Xiong Y., Li M., Zhang J., Wang J. (2020). Cuins/zns quantum dots modified intraocular lens for photothermal therapy of posterior capsule opacification. Exp. Eye Res..

[68] Syed Hussain S., Donempudi S., Tammishetti S., Garikapati K.R., Bhadra M.P. (2018). Cell adhesion resistant, uv curable, polymer zinc oxide nanocomposite materials for intraocular lens application. Polym. Adv. Technol..

[69] Tan X., Zhan J., Zhu Y., Cao J., Wang L., Liu S., Wang Y., Liu Z., Qin Y., Wu M. (2017). Improvement of uveal and capsular biocompatibility of hydrophobic acrylic intraocular lens by surface grafting with 2-methacryloyloxyethyl phosphorylcholine-methacrylic acid copolymer. Sci. Rep..

[70] Viveiros M.M., Soares R.T., Omodei M.S., Rainho C.A., Padovani C.R., Cruz N., Schellini S.A., Rodrigues A.C. (2015). Adhesion study of cultured human lens capsule cells on hydrophilic intraocular lenses coated with polyethylene glycol. J. Cataract Refract. Surg..

[71] Wang B., Lin Q., Jin T., Shen C., Tang J., Han Y., Chen H. (2015). Surface modification of intraocular lenses with hyaluronic acid and lysozyme for the prevention of endophthalmitis and posterior capsule opacification. RSC Adv..

[72] Xu X., Tang J.-M., Han Y.-M., Wang W., Chen H., Lin Q.-K. (2016). Surface pegylation of intraocular lens for pco prevention: An in vivo evaluation. J. Biomater. Appl..

[73] Zhang H., Zhang X., Kuang Z., Jin Y., Pang S., Wang Y., Lin D., Chen H., Qian S., Wang B. (2020). Bionic antibacterial modification of iol through si-raft polymerization of p(toeac-co-mpc) brushes to prevent pco and endophthalmitis. Polym. Test..

[74] Zheng Z., Wang Y., Jiao Y., Zhai Z. (2016). Biological performance of functionalized biomedical polymers for potential applications as intraocular lens. J. Biomed. Mater. Res. A.

[75] Matsushima H., Iwamoto H., Mukai K., Obara Y. (2006). Active oxygen processing for acrylic intraocular lenses to prevent posterior capsule opacification. J. Cataract Refract. Surg..

[76] Krall E.M., Arlt E.-M., Jell G., Strohmaier C., Bachernegg A., Emesz M., Grabner G., Dexl A.K. (2014). Intraindividual aqueous flare comparison after implantation of hydrophobic intraocular lenses with or without a heparin-coated surface. J. Cataract Refract. Surg..

[77] Spalton D.J., Russell S.L., Evans-Gowing R., Eldred J.A., Wormstone I.M. (2014). Effect of total lens epithelial cell destruction on intraocular lens fixation in the human capsular bag. J. Cataract Refract. Surg..

[78] Nassiri S., Hwang F.S., Kim J., LeClair B., Yoon E., Pham M., Rauser M.E. (2019). Comparative analysis of intravitreal triamcinolone acetonide–moxifloxacin versus standard perioperative eyedrops in cataract surgery. J. Cataract Refract. Surg..

[79] Lipnitzki I., Eliahu S.B., Marcovitz A.L., Ezov N., Kleinmann G. (2014). Intraocular concentration of moxifloxacin after intracameral injection combined with presoaked intraocular lenses. J. Cataract Refract. Surg..

[80] Liu Y.-C., Wong T.T., Mehta J.S. (2013). Intraocular lens as a drug delivery reservoir. Curr. Opin. Ophthalmol..

